# Repurposing Butenafine as An Oral Nanomedicine for Visceral Leishmaniasis

**DOI:** 10.3390/pharmaceutics11070353

**Published:** 2019-07-20

**Authors:** Adriana Bezerra-Souza, Raquel Fernandez-Garcia, Gabriela F. Rodrigues, Francisco Bolas-Fernandez, Marcia Dalastra Laurenti, Luiz Felipe Passero, Aikaterini Lalatsa, Dolores R. Serrano

**Affiliations:** 1 Laboratory of Pathology of Infectious Diseases (LIM-50), Medical School, University of São Paulo, Avenida Dr. Arnaldo 455, 01246903 Cerqueira César, SP, Brazil; 2 Departament of Pharmaceutics and Food Technology and Instituto Universitario de Farmacia Industrial (IUFI), School of Pharmacy, University Complutense, Avenida Complutense, 28040 Madrid, Spain; 3 Departament of Microbiology and Parasitology, School of Pharmacy, Universidad Complutense de Madrid, Plaza Ramón y Cajal s/n, 28040 Madrid, Spain; 4 Institute of Biosciences, São Paulo State University (UNESP), São Vicente. Praça Infante Dom Henrique, s/n, 11330-900 São Vicente, SP, Brazil; 5 Institute of Biomedical and Biomolecular Sciences, School of Pharmacy and Biomedical Sciences, University of Portsmouth, White Swan Road, Portsmouth PO1 2 DT, UK

**Keywords:** butenafine, SNEDDS, solid SNEDDS, spray drying, leishmaniasis, design of experiments

## Abstract

Leishmaniasis is a neglected tropical disease affecting more than 12 million people worldwide, which in its visceral clinical form (VL) is characterised by the accumulation of parasites in the liver and spleen, and can lead to death if not treated. Available treatments are not well tolerated due to severe adverse effects, need for parenteral administration and patient hospitalisation, and long duration of expensive treatments. These treatment realities justify the search for new effective drugs, repurposing existing licensed drugs towards safer and non-invasive cost-effective medicines for VL. In this work, we provide proof of concept studies of butenafine and butenafine self-nanoemulsifying drug delivery systems (B-SNEDDS) against *Leishmania infantum*. Liquid B-SNEDDS were optimised using design of experiments, and then were spray-dried onto porous colloidal silica carriers to produce solid-B-SNEDDS with enhanced flow properties and drug stability. Optimal liquid B-SNEDDS consisted of Butenafine:Capryol 90:Peceol:Labrasol (3:49.5:24.2:23.3 *w*/*w*), which were then sprayed-dried with Aerosil 200 with a final 1:2 (Aerosil:liquid B-SNEDDS *w*/*w*) ratio. Spray-dried particles exhibited near-maximal drug loading, while maintaining excellent powder flow properties (angle of repose <10°) and sustained release in acidic gastrointestinal media. Solid-B-SNEDDS demonstrated greater selectivity index against promastigotes and *L. infantum*-infected amastigotes than butenafine alone. Developed oral solid nanomedicines enable the non-invasive and safe administration of butenafine as a cost-effective and readily scalable repurposed medicine for VL.

## 1. Introduction

Leishmaniasis is an infectious disease caused by parasites belonging to the *Leishmania* genus. The prevalence of leishmaniasis exceeds 12 million cases, and it is endemic in 98 countries in five continents. *Leishmania* parasites are transmitted by insect vectors from the genus *Lutzomyia* sp. or *Psychodopygus* sp. in the New World and *Phlebotomus* sp. in the Old World [1,2]. Leishmaniasis presents in the cutaneous (CL) and visceral (VL) leishmaniasis forms, depending on the type of host immune response and infecting parasite species [3,4]. In the New World, parasites of the subgenus *Vianna* cause only CL and mucocutanoues leishmaniasis (MCL) while parasites of the subgenus *Leishmania* are responsible for CL and VL [5]. VL is a chronic disease caused by *L. (L.) infantum* and *L. (L.) donovani* species [6,7,8] residing in host macrophages, mainly from spleen, liver, bone marrow, and lymph nodes, and is characterized by prolonged fever, hepatosplenomegaly, lymphadenopathy, anemia with leukopenia, hypergammaglobulinemia and hypoalbuminemia, weight loss, edema, and a debilitating state leading to weakening and ultimately death if untreated [6].

VL treatment mainstays involve pentavalent antimonials (SbV) and amphotericin B (AmB) as the first line in the developing and developed world, respectively [9]. Although they are both highly efficacious in vivo, SbV are linked to severe and frequent side effects, limiting their use [9], combined with a high rate of clinical resistance [10]. AmB, on the other hand, has shown limited resistance, but its clinical use is limited by the high cost, especially for safer lipidic formulations, such as Ambisome^®^, thermal instability, and nephrotoxicity [11,12]. Miltefosine, the only oral VL licensed treatment (licensed in 2003 in India), has several limitations as monotherapy, as its activity is highly dependent on the clinical form of leishmaniasis and the parasite strain, and patients frequently experience severe gastrointestinal disorders. In addition, it is teratogenic, and thus cannot be used in women of child-bearing age [13,14]. Miltefosine has a long elimination half-life (seven days) and a narrow therapeutic index, characteristics that limit the administered dose, which can lead to subtherapeutic levels over several weeks, encouraging the emergence of resistance [12,14,15]. Indeed, resistance has been reported in India (a country that alone accounts for 50% of the VL worldwide burden) and France [12]. Thus, available medicines for VL are outdated, impractical, insufficiently efficacious, or subject to resistance and unacceptable toxicities. Ideal treatments for VL should be able to possess greater than 95% efficacy, be orally administered, be stable in a tropical environment, be affordable, and require fewer than 11 days of treatment with an excellent side-effect profile [12,16].

Repurposing drugs is a strategy that has been crowned with success, as repurposed drugs have made up a third of all new commercially and clinically used drug treatments since 2009 [17,18]. Current VL drugs are interesting examples of repurposed drugs, since SbV, AmB, and miltefosine were originally used as an emetic, antifungal, and anticancer drug, respectively [14,19]. Butenafine is an allylamine drug, commonly employed in the treatment of fungal skin infections, such as ringworm, athlete’s foot, jock itch, and pityriasis. Currently, it is only commercialised as a 1% cream for topical use. We have shown recent reports on how butenafine repositioning was effective in vitro against *L. (L.) amazonensis* and *L. (V.) braziliensis*, ethiological agents of CL and MCL. Furthermore, parasite treated with butenafine showed morphological alterations that resembled programmed cell death, which is attributed to the blockage of the biosynthesis of ergosterol [20]. Butenafine has limited oral bioavailability, with 1.5–3% of the oral dose being recovered in the plasma an hour after a single oral dosing of radiolabeled butenafine (0.2 mg/kg) in rats [21]. Butenafine is highly metabolized in the liver (methylation, dealkylation, and hydroxylation) and only 0.03% of the oral dose has been recovered intact from the plasma after 4 h [21]. This concurs with levels of its major metabolite (1-napthoic acid) in the plasma, which ranged between 1%–100% of administered parent drug dose [21].

Here, we are reporting the development of an oral butenafine nanomedicine able to enhance its aqueous solubility, maintaining a solubilized state in the gastrointestinal to allow for enhanced oral absorption, in order to target the liver and spleen (i.e., organs where the *Leishmania* parasite resides in high concentration). We have shown that SNEDDS are able to enhance the oral bioavailability of poorly soluble drugs and enable therapeutic concentrations to be delivered in the liver and spleen [12]. Thus, we hypothesised that if butenafine is formulated with GRAS (Generally Regarded as Safe) excipients with known activity against different *Leishmania* strains [22], we can develop butenafine-loaded SNEDDS (B-SNEDDS) and solid SNEDDS (solid B-SNEDDS) with enhanced activity, as well as being able to maintain butenafine’s oral solubilisation capacity in the gastrointestinal tract. To ensure that a stable, and ideally solid, cost-effective formulation is available, we used design of experiments (DoE) studies to prepare butenafine SNEDDS colloidal silicon dioxide spray-dried particles that can be easily compressed into cost-effective, easily scalable, solid dosage forms of a repurposed drug for VL.

## 2. Materials and Methods

### 2.1. Materials

Butenafine hydrochloride (purity ≥ 98%) was purchased from Cayman Chemical Co. (Michigan, MI, USA). SNEDDS excipients (Capryol 90 (propylene glycol monocaprylate), Labrafil M 1944 CS (oleoyl polyoxyl-6 glycerides), Labrasol (caprylocaproyl polyoxyl-8 glycerides), and Peceol (glyceryl monooleate)) were kindly donated by Gattefosse (Saint-Priest Cedex, France). Two Aerosil silicon dioxide excipients were used as inert solid carriers: Aerosil R972 from Degussa (Frankfurt, Germany) and Aerosil 200 from Evonik Industries (Darmstadt, Germany). All other chemicals were of ACS reagent grade (Sigma Aldrich, Madrid, Spain) and were used as supplied. Solvents were of HPLC grade (Fisher, Madrid, Spain).

### 2.2. Solubility Studies of Butenafine

An excess quantity of butenafine was added to each of the excipients used in the preparation of SNEDDS, and the mixture was shaken (300 rpm) overnight at 25 °C in triplicate. The mixtures were centrifuged at 3000 rpm for 5 min, and the supernatant (0.1 mL) was diluted with 10 mL of methanol. The absorbance was measured in a spectrophotometer (JASCO V-730 spectrophotometer Madrid, Spain) at 220 nm to determine the solubility of butenafine. A calibration curve was performed previously in methanol to establish the linearity between concentration and absorbance at 220 nm.

### 2.3. Pseudo-Ternary Phase Diagrams

Ternary phase diagrams were constructed to study the phase behaviour of oils/surfactants over the whole concentration range. The existence of a microemulsion (type II) region within this diagram was observed visually. A D-Optimal design was developed using different mixtures of Capryol 90, Labrasol, and Peceol by using Design Expert software (State Ease, Minneapolis, MN, USA). Mixtures were vortexed for 5 min. The particle size distribution (PSD) was measured after dilution in deionised water (1:1000 *v*/*v*) in a Microtrac Zetatrac (Microtrac, Montgomeryville, PA, USA). The optimal excipient combination leading to the smallest PSD upon dilution was selected, in order to solubilise the drug and perform further experiments.

### 2.4. Preparation of Liquid B-SNEDDS Formulations

Based on solubility and phase diagram studies, Labrasol, Capryol 90, and Peceol were selected as a high-hydrophilic-lipophilic balance (HLB) surfactant, a medium-HLB surfactant, and an oil, respectively. Optimal SNEDDS were prepared combining the three as Capryol 90:Labrasol:Peceol (51:24:25 *w*/*w*). Butenafine (30 mg/g) was solubilised in the resulting excipient mixture, which was stirred overnight in order to obtain a homogenous isotropic mixture.

### 2.5. Preparation of Solid B-SNEDDS Formulation

A mini-spray dryer (Büchi B-191) was employed for the preparation of solid B-SNEDDS. Aerosil 200 (hydrophilic fumed amorphous silica, 5–50 nm (Evonik industries, Darmstadt, Germany)) or Aerosil R972 (hydrophobic fumed amorphous silica, 16 nm (Degussa AG, Frankfurt, Germany)) were used as inert carriers. A carrier (1 g) was dispersed in 100 mL of ethanol by magnetic stirring, after which liquid B-SNEDDS formulation (0.5 g) was mixed. The resulting suspension was delivered to a two-fluid nozzle (0.7 mm nozzle tip and a 1.5 mm diameter nozzle screw cap) using a peristaltic pump, at a speed of 10% (5 mL/min). Compressed air (2 bars) was used as the drying gas in a co-current mode, with the aspirator capacity set to maximum (100%). The flow-meter for the standard two-fluid nozzle was set to 600 NL/h. The inlet temperature was fixed at 62 °C, and the outlet temperature varied between 32 and 36 °C. After spray-drying, the dry powder was collected from the collector vessel, and the yield of the process was quantified using the following equation:

(1) $$Yield\;\left(\%\right)=\frac{Collected\;mass\;after\;spray\;drying\;\left(mg\right)}{Total\;mass\;spray\;dried\;\left(mg\right)}\;\times 100$$

A 2^2^ DoE was performed to understand and optimise key formulation parameters affecting the preparation of solid B-SNEDDS. The surface properties of silica used, i.e., type of Aerosil (200 or R972), as well as the weight ratio between Aerosil and liquid B-SNEDD (1:2 or 1:3 *w*/*w*) on the physicochemical properties of the spray-dried product were investigated. Five different responses were studied: (i) powder flow, (ii) yield, (iii) drug loading, (iv) particle size upon dilution (1:1000 *w*/*w*), and (v) the percentage of drug release at 60 min in simulated gastric fluid (500 mL buffer solution of pH 1.2).

### 2.6. Characterisation of the Solid B-SNEDDS

#### 2.6.1. Powder Flow: Angle of Repose (AoR) Measurements

The angle of repose (AoR) was determined according to the United State Pharmacopeia (<1174> Powder Flow) by using the fixed height funnel method (i.e., by measuring the cone height versus the base, formed by the powder falling through a plastic funnel placed 10 cm from the table surface until a stable cone was formed) [23]. AoR measurements were carried out by passing 500 mg of solid B-SNEDD in triplicate. After the powder was deposited on the surface, the height of the powder cone (*h*) and the radius of the base (*r*) were measured. The inclination of the cone created between the powder and the surface (α, AoR) was calculated using the following equation:

(2) $$\tan\left(\alpha \right)=\frac{h}{r}$$

#### 2.6.2. Particle Size Measurements

The particle size of both liquid B-SNEDDS and solid B-SNEDDS were determined at 25 °C after 1:1000 *w*/*w* dilution in deionised water. Solid B-SNEDDS were centrifuged (9000 rpm, 5 min) prior to measurements, in order to precipitate the insoluble carrier. The mean size (*n* = 3) by volume (nm) was measured using a Microtrac Zetatrac (Microtac Inc., Montgomeryville, PA, USA), with an internal probe ranging from 0.0008 to 6.5 µm [24].

#### 2.6.3. Release Studies

Release studies of solid B-SNEDDS (500 mg) containing 10 mg of butenafine were performed at 37 °C for 1 h in simulated gastric fluid (SGF; 500 mL buffer solution of pH 1.2), followed by a second consecutive hour in simulated intestinal fluid (SIF; 400 mL buffer solution of pH 6.8) in a calibrated dissolution apparatus (Erweka type DT80, Erweka, Heusenstamm, Germany). The SGF and SIF were prepared as described in the USP [25]. Based on predicted water solubility values (7.5 × 10^−5^ mg/mL, according to the Drug Bank Database [26]), release studies were performed in non-sink conditions, with the aim of testing a relevant pharmacological drug dose (10 mg). At different time points (5, 10, 15, 30, 45, 60, 90, and 120 min), a 2 mL sample was withdrawn, filtered through a Millipore Millex PTFE membrane filter (0.45 µm), diluted 1:2 with acetonitrile, and injected in the HPLC. Butenafine concentration was quantified using an HPLC equipped with a Jasco PU-1580 pump, a Jasco AS-2050 Plus autosampler, and a Jasco UV-1575 UV-visible detector. Integration of the peaks was performed using the Borwin 1.5 software. Butenafine was separated on an Agilent Eclipse XDB-Phenyl reverse-phase column (250 mm × 4.6 mm, 5 µm). The mobile phase consisting of methanol/water (78:22 *v*/*v*) was pumped at 1.4 mL/min, and a sample injection volume of 20 µL was used. The column temperature was kept at 25 °C, and the detector was set at 220 nm. Butenafine concentrations were calculated from a linear regression calibration curve between 100.0–0.1 µg/mL.

#### 2.6.4. Drug Loading

Solid B-SNEDD formulations (10 mg) were dissolved in 0.5 mL of dimethyl sulfoxide (DMSO), vortexed for 5 min, and then diluted in acetonitrile (1:40 *v*/*v*) prior to drug quantification by the above-described HPLC method.

#### 2.6.5. Morphological Analysis

The morphology of solid B-SNEDDS was examined using a scanning electron microscope (JEOL JSM 6335F Ltd., Akishima, Japan) at 5 kV. The samples were fixed on a brass stub using double-sided adhesive tape, and vacuum-coated with gold (Au) for 180 s (coater: Q150R S, Quorum, Lewes, East Sussex, United Kingdom).

#### 2.6.6. Tabletting and Hardness

In order to investigate the compression of the solid B-SNEDDS without the addition of any other excipient, solid B-SNEDDS (500 mg) were compressed using a Perkin Elmer hydraulic press (Waltham, MA, USA) and a 13 mm punch, and die set under different pressures of 0.5, 1.0, 3.0 or 5.0 tonnes for 15 s. Hardness was undertaken according to the European Pharmacopeia [27], using a Pharma Test PTB 311 instrument (Pharma Test, Hainburg, Germany). Tablets (*n* = 3) were individually evaluated, and the mean value of the force in Newtons (N) was reported.

### 2.7. In Vitro Efficacy and Toxicity Studies

#### 2.7.1. Parasites and Cell Lines

The parasites were kindly provided by Prof. Dr. Fernando Tobias Silveira from the cryobank of the Leishmaniasis Laboratory of Prof. Dr. Ralph Laison, Department of Parasitology, Ministry of Health, Evandro Chagas Institute (Belem, Para, Brazil). They were identified using monoclonal antibodies and isoenzyme electrophoretic profiles at the Leishmaniasis Laboratory of the Evandro Chagas Institute. The *Leishmania* species used was *L. (L.) infantum* (MHOM/BR/72/46). Parasites in a late log stage were used for all experiments. Parasites were maintained in Schneider’s Medium (SigmaAldrich, Madrid, Spain), supplemented with 10% heat-inactivated fetal bovine serum, 50,000 IU/mL penicillin, and 50 μg/mL streptomycin.

BALB/c mice, 6 weeks old, were obtained from the Medical School of the University of São Paulo, Brazil, in order to collect peritoneal macrophages to perform the in vitro test against intracellular *Leishmania* amastigotes. To obtain the macrophages, the animals were anaesthetized with thiopental (1 mg/200 mL) and euthanized. This study was carried out in strict accordance with the recommendations detailed in the Guide for the Care and Use of Laboratory Animals of the Brazilian National Council of Animal Experimentation (http://www.cobea.org.br). The protocol was approved by the Ethics Committee of Animal Experiments of the Institutional Committee of Animal Care and Use at the Medical School of Sao Paulo University (CEUA-FMUSP number 098/17).

#### 2.7.2. In Vitro Promastigote Efficacy and Cytotoxicity

Promastigote forms of *L. (L.) infantum* (2 × 10^7^ promastigotes/well) were incubated in a 96-well culture plate in RPMI 1640 medium, with pH 4.2 and drugs in a range of 0.01 to 400 μΜ. The negative control group was cultivated in medium and vehicle solution (PBS plus 1% DMSO). The parasites were incubated for 48 h at 25 °C, and washed with 200 μL of sodium chloride 0.9% (*w*/*v*) three times with centrifugation at 3000 rpm for 10 min at 4 °C, followed by the addition of MTT (3-(4,5-dimethylthiazol-2-yl)-2,5-diphenyltetrazolium bromide) (9.6 μM). Four hours later, 50 μL of 10% sodium dodecyl sulphate (SDS) was added to each well. The plates were further incubated for 18 h and read in an ELISA reader (Labsystems Uniscience Multiskan EX, Miami, FL, USA) at 595 nm. Effective concentration 50% (EC_50_) was estimated using Graph Pad Prism 5.0 software (GraphPad Software, San Diego, CA, USA).

Approximately 5 × 10^5^ peritoneal macrophages from BALB/c mice were cultured in RPMI 1640 medium, with the drugs in a range of 0.01 to 400 μΜ, in 96-well plates. As a negative control, macrophages were cultivated with vehicle solution. After 48 h, cell viability was analysed by the MTT method. Cytotoxic concentration 50% (CC_50_) was estimated with Graph Pad Prism 5.0 software.

The selectivity indexes (SI) were calculated using the ratio CC_50_/EC_50_ toward promastigote (SIp) or amastigote (SIa) forms. EC_50_ represents the concentration of the formulation that produced a 50% reduction in parasites, while CC_50_, represents the concentration of the formulation that produced a 50% reduction of cell viability in treated culture cells with respect to untreated ones.

#### 2.7.3. Macrophage Infection and Treatments

Peritoneal macrophages from BALB/c mice (5 × 10^5^ macrophages) were cultivated in round cover slips in a 24-well plate, followed by infection with *L. (L.) infantum* promastigotes at a ratio of 10 parasites per 1 peritoneal macrophage. Plates were incubated at 5% CO_2_ at 37 °C. After 24 h of the initial infection, drugs were added at 25, 50, or 100 μM. Round cover slips from each experimental time point were dried at room temperature, fixed in methanol, and stained by Giemsa 5% (two drops). The infection index (II) was then estimated according to Passero et al. [28], using the following equation:

(3) $$II=\%\;infected\;macrophages\;\times \;\frac{Internalized\;amastigotes}{Macrophages}$$

### 2.8. Statistical Analysis

Statistical analyses were performed via a one-way ANOVA test using Minitab 15 (Minitab Ltd., Coventry, UK), followed by Tukey’s test. Statistical significance was set at *p* < 0.05. Data was plotted using Origin X9 (Northampton, UK).

## 3. Results

### 3.1. Solubility Studies of Butenafine

The solubility of butenafine was tested in four different excipients (Peceol, Capryol 90, Labrasol, and Labrafil M1944CS) that are commonly utilized in the development of lipid-based formulations and have been shown to possess efficacy against leishmaniasis [22] (Table 1). Butenafine was more soluble in excipients with oleic acid lipids or triglycerides, i.e., Peceol and Labrafil M 1944 CS, followed by lower HLB excipients such as Capryol 90. Due to the low miscibility between Peceol and Labrafil, and the higher butenafine solubility in Peceol, Peceol was chosen as the oil phase to be combined with Capryol and Labrasol for the pseudo-ternary diagram and identification of the optimal composition (Table 2) able to yield microemulsions (type II) upon aqueous dilutions.

### 3.2. Pseudo-Ternary Phase Diagrams and Preparation of Liquid B-SNEDDS Formulations

**Figure 1:**
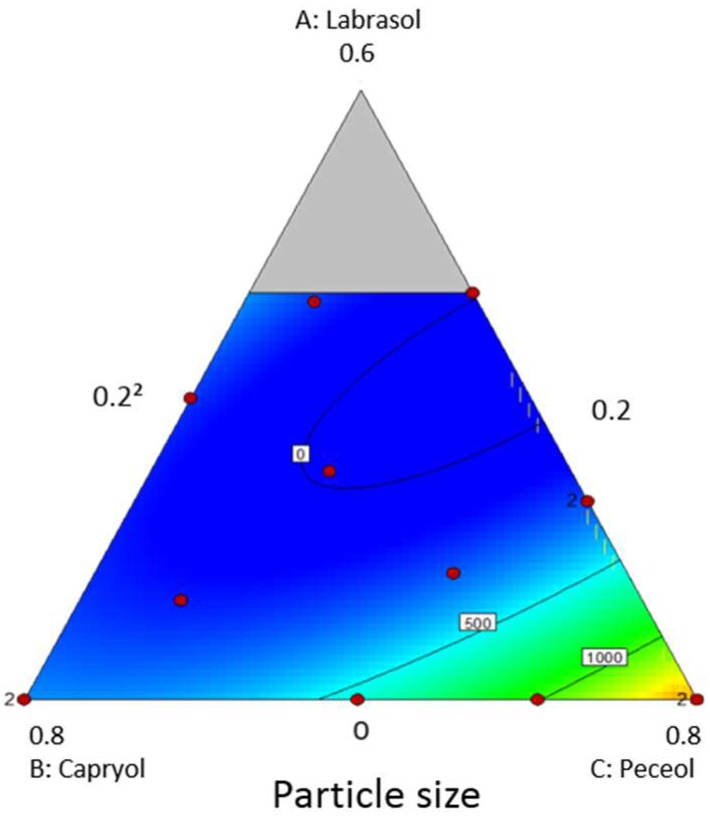
Pseudo-ternary phase diagram of SNEDDS. In the white rectangles is indicated the particle size of each corresponding composition. The blue areas indicate a lower particle size, while yellow and red areas indicate larger sizes.

A ternary phase diagram (Table 2 and Figure 1) was constructed to study the phase behaviour of oil/surfactants over the whole concentration range. Particle size expressed in numbers led to a better predictive model with a higher R^2^ than the one using values expressed in volume. The blue region indicates the self-nanoemulsifying region, with a lower particle size. The particle size optimisation studies suggested two optimal excipient combinations with the following composition:

(1) Combination A: Peceol:Capryol:Labrasol mixed at 27:58:15 *w*/*w*
(2) Combination B: Peceol:Capryol:Labrasol mixed at 25:51:24 *w*/*w*

Validation studies were performed by preparing the suggested optimal mixtures, followed by measuring the particle size. The resulted particle size was 159.7 nm for combination A, and 88.4 nm for combination B. Based on these results, combination B was selected for further development of solid-B-SNEDD formulations.

### 3.3. Yield of Solid B-SNEDDS

Four different solid B-SNEDDS were manufactured utilising the vehicle mixture (combination B) above described. Two different hydrophobic, fumed silica carriers (Aerosil 200 or R972) were combined in two ratios (1:2 and 1:3 *w*/*w*), with the optimised liquid B-SNEDDS and the resulting four formulations coded as F1 to F4, respectively (Table 3). When SNEDDS were adsorbed on Aerosil 200 at a 1:2 ratio (F1), a higher yield was obtained, which was able to carry higher amounts of liquid B-SNEDDS (1:3 ratio). Hydrophobic fumed silica resulted in a poorer yield, indicating that SNEDDS adsorbed easier towards a hydrophilic surface, suggesting a core shell particle structure for liquid B-SNEDDS, with Labrasol being orientated towards the surface, and likely to interact more strongly with hydrophilic silica surfaces. However, this stronger interaction of labrasol with hydrophilic silicas has been linked previously to poorer disintegration times, which should be taken into account later when manufacturing solid dosage forms, such as tablets [30].

All solid B-SNEDDS had an AoR below 20°, indicative of excellent flow properties [31,32] with near maximal drug loading (Table 3). Particle size measurements confirmed microemulsion regions with a size well below 300 nm. F4 yielded particles that either had not been able to re-form after adsorption on silica, or were unstable after the 1:1000 dilution, and thus were considered not appropriate for further development.

### 3.4. Release Studies

**Figure 2:**
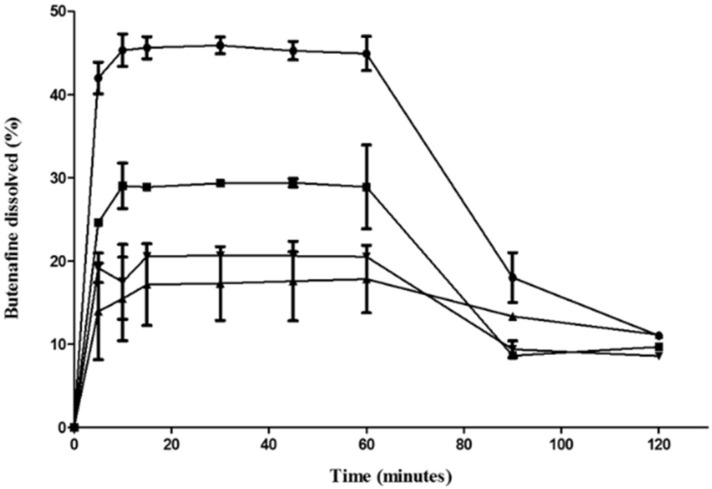
Release of solid B-SNEDDS formulations in simulated gastric fluid (SGF) (pH 1.2, first 60 min) and simulated intestinal fluid (SIF) (pH 6.8) thereafter (mean % ± SD). -●- F1, -■-F2, -▲- F3, -▼- F4.

Hydrophilic fumed silica particles indicated a greater and faster release of butenafine nanoemulsions type II (F1 > F2 > F3~F4) (Figure 2). Increasing the pH from 1.2 to 6.8 to mimic the intestinal pH resulted in a sharp precipitation, which can be linked with a spring–parachute effect. This can explain the low absorption of butenafine orally [21], as that only allows for absorption in the upper part of the gastrointestinal tract [33,34]. Butenafine is more soluble in acidic pH as a hydrochloride salt, while its base (pKa: 9.23) has limited water solubility (<100 ng/mL) [35]. Formulation F1 allows for higher solubilised levels up to 90 min (*p* < 0.05; one-way ANOVA), which is critical for enhancing oral absorption.

### 3.5. Morphological Analysis

**Figure 3:**
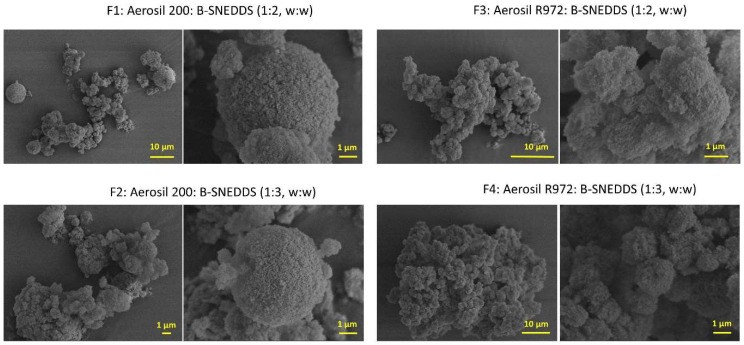
Scanning electron microscope (SEM) micrographs of solid B-SNEDDS F1, F2, F3, and F4 formulations at two different magnifications.

The morphology of the four solid B-SNEDDS formulations was observed by scanning electron microscopy (Figure 3). At higher magnification, the large surface porosity of both silicon dioxide carriers can be observed. This explains the high amount of liquid SNEDDS that is able to be adsorbed on to the solid carriers, up to three times their own weight. However, agglomeration between carrier particles was more noticeable in those formulations containing a higher ratio of liquid SNEDDS (F2 and F4), which can explain the hindered release observed. In the F3 and F4, both containing the most hydrophobic carrier (Aerosil R972), it was observed that even at the lower ratio of liquid-SNEDDS (1:2 *w*:*w*), the aggregation between particles is still evident to some extent.

### 3.6. Tableting and Hardness

Amongst all the solid B-SNEDDS, only the F1 formulation was able to be compacted with adequate hardness without the incorporation of other excipients. We should bear in mind that the addition of other excipients will further dilute the butenafine dose per tablet size, which currently is 10 mg/500 mg tablet in powder form (Table 4). The addition of microcrystalline cellulose is likely to allow other solid SNEDDS to be formulated with appropriate hardness.

### 3.7. In Vitro Efficacy and Cytotoxicity

Miltefosine was active against both promastigote and amastigote forms of *L. (L.) infantum*, exhibiting an EC_50_ of 17.9 ± 0.9 and 13.7 ± 0.7µM, respectively, as well as a mild cytotoxicity (CC_50_ of 126.3 ± 3.5 µM) leading to a selectivity index towards promastigotes forms (SIp) of 7.0 and a selectivity index towards amastigotes forms (SIa) of 9.2 (Table 5). These values are similar to previous documented studies [36]. Free butenafine showed lower efficacy than miltefosine (Table 5), with an SI close to the unit indicating poor selectivity for the parasites over the macrophages. Amongst the four solid B-SNEDDS, F1 and F4 showed better efficacy against promastigotes (*p* < 0.05), while Aerosil R972 formulations (F3 and F4) demonstrated lower cytotoxicity and higher efficacy against amastigotes (*p* < 0.05), but whether this is driven by the excipient is not clear, and further studies are needed. In any case, except for F2, formulating butenafine as SNEDDS has improved its selectivity index, making it less toxic and more active than the free drug.

## 4. Discussion

Butenafine, a benzylamine derivative structurally similar to terbinafine, possesses antifungal activity, attributed to its ability to directly cause damage on fungal cell membranes by disrupting the early stages of ergosterol biosynthesis via inhibition of the enzyme squalene epoxidase [37]. This enzyme converts squalene to lanosterol, and leads to the accumulation of squalene [38]. Inhibition of squalene epoxidase suppresses the biosynthesis of ergosterol, an essential lipid of fungal and *Leishmania* cell membranes [39].

Therefore, butenafine is known for its antifungal effects in infections caused by *Tinea pedis, Tinea corporis,* and *Tinea cruris* [40]. We have recently demonstrated the leishmanicidal effect of butenafine against promastigote and amastigote forms of *L. (L.) amazonensis* and *L. (V.) braziliensis* [20]. Considering the difficulties related to the treatment of leishmaniasis, such as drug relapses, toxicity, hospitalization, and parenteral administration, the development of an oral, safe, multispecies, and effective medicament could revolutionise VL treatment. Butenafine is a multispecies drug, able to be effective against a range of parasite strains, such as *L. (L.) amazonensis, L. (V.) braziliensis*, and now in this work, it has been demonstrated that is also active against *L infantum*. However, due to its poor water solubility and precipitation in the gastrointestinal tract, no oral formulations of butenafine have been licensed.

In order to pave the way for the market of oral medicines against VL, the selection of excipients that can promote oral bioavailability and safety, as well as elicit a synergistic effect against Leishmania parasites without compromising safety is critical. Peceol, Labrasol, and Capryol 90 have been selected as GRAS excipients to formulate butenafine SNEDDS, able to enhance the drug solubilisation capacity in the gastrointestinal tract and oral bioavailability [41]. Also, due to the proven efficacy of those selected excipients against several strains of Leishmania parasites, a synergistic effect with butenafine can be expected [22].

Pre-formulation studies have indicated that butenafine is more soluble in C18 lipids and amphiphiles, and these were thus selected for forming the oil component of SNEDDS (Peceol). We decided to combine these with the medium, as well as short chain fatty acids esters (Capryol 90) and triglycerides (Labrasol) that we have recently shown to possess antileishmanial activity. The high reported activity of lauric acid and labrasol against *Leishmania* parasites can be attributed to their ability to selectively permeate the cell membrane of parasites, resulting in rapid and considerable membrane damage and the loss of cellular potassium and magnesium [22]. SNEDDS were optimised towards a minimised droplet particle size (~100 nm) for several reasons. Nanoemulsions (type II microemulsions) are more stable than microemulsions, in terms of droplet flocculation and coalescence (Ostwald ripening) [42]. The smaller the particle size, the higher the permeability across the intestinal mucus brush border layer and cell membranes [43]. Additionally, small droplet sizes (~90 nm) provide a large interfacial surface area for drug release and absorption across the intestinal cells [44]. Recent reports suggest that a particle size between 100–500 nm is optimal for lymphatic uptake via the gastrointestinal lymphatic system, but at a slower rate than particles sized 50–100 nm [45,46].

In order to reduce costs, increase the chemical stability, and improve patient compliance, B-SNEDDS was transformed into a solid free-flowing powder by spray-drying, which can be directly compressed. Fumed amorphous silica (Aerosil) are known to possess enhanced surface area for adsorption (Aerosil 200: 200 ± 25 m^2^/g and Aerosil R972: 110 ± 20 m^2^/g). Both silicas were able to adsorb three times their own weight in SNEDDS, resulting in powders with excellent flow properties, as indicated by AoR studies (<20°) [30]. The higher porosity and hydrophilic surface of Aerosil 200 carriers explain the higher drug release and yield, respectively. Loading was higher with Aerosil 200, which indicates a core-shell morphology of the SNEDDS droplet, with polyethylene glycol chains stabilising the droplet surface and enabling higher interactions with the hydrophilic surface of the silica [47,48]. Aerosil R972 carriers hinder the release of butenafine, possibly due to stronger hydrophobic interactions; however, this is likely to limit oral bioavailability. Additionally, particles prepared with Aerosil R972, with high SNEDDS loading when dispersed in water, are unable to yield an emulsion, indicating that droplets are not able to re-form or are unable to maintain their morphology, and become unstable after a 1:1000 dilution. The latter, combined with limited release from these solid B-SNEDDS, makes them less likely to be able to enhance oral bioavailability. Interestingly, though, these particles have a significantly impact on the in vitro efficacy against *Leishmania* promastigotes and amastigotes. Even though butenafine release from F3 and F4 formulations was low at physiological pH, these formulations possessed enhanced efficacy and selectivity, with no higher cytotoxicity on macrophages. Enhanced surface hydrophobicity can trigger macrophage uptake compared to particles with a hydrophilic surface [49]. However, when the results are taken together, formulation F1 is more likely to be a promising solid nanomedicine for VL, as it maintains characteristics for enhanced solubilisation and oral uptake, as well as presented antileishmanial activity in *L. (L.) infantum* promastigotes and amastigotes.

Limited studies are available in the literature for understanding the ability of solid carriers to load SNEDDS. Preparing a conventional solid dosage form of SNEDDS remains challenging, as tablets can be friable if a low compression force is used, or allow for SNEDDS leakage if a high compression force is used. Tablets prepared with Labrasol and silica (Neusilin US2, a synthetic, amorphous form of magnesium aluminometasilicate) have demonstrated an enhanced disintegration time and the need for a disintegrant [32]. Our solid B-SNEDDS were able to load high SNEDDS quantities, while being free-flowing and easily compacted into the appropriate hardness tablets. Smith et al. [12] have demonstrated that liquid SNEDDS can be transformed into solid tablets with good release properties when the ratio between liquid to solid was 1:3. In our work, we have shown that even a much greater ratio of liquid to solid (2:1) can still be compacted without SNEDDS leakage and maintain a homogenous matrix for compaction. However, further studies are required to investigate the manufacturing of these free-flowing powders into tablets that meet the USP Pharmacopeia requirements.

## 5. Conclusions

Manufacturing of cost-effective solid dosage forms of reformulated drugs is an ideal strategy to speed up the development of novel medicines for VL and other neglected diseases. We have demonstrated for the first time the efficacy of butenafine for VL, while developing an easily scalable preparation from GRAS excipients of solid dosage forms of butenafine, able to maintain its solubilisation capacity in the gastrointestinal tract. SNEDDS were optimised for drug loading (30 mg/g) and particle size, and we have demonstrated the ability of a microemulsion to be formed after release from fumed silica particles. Solid SNEDDS demonstrated excellent flow properties, and were able to be compressed in adequate hardness tablets. They also demonstrated antileishmanial activity in *L. (L.) infantum* promastigotes and amastigotes, which indicated their potential as solid nanomedicines for the treatment of VL.

## Figures and Tables

**Table 1:** Solubility of butenafine in various vehicles at 25 °C (*n* = 3).

Vehicle	Butenafine (mg/g)	HLB *
Peceol	24.68 ± 0.21	1
Capryol 90	15.79 ± 1.18	5
Labrasol	13.03 ± 3.94	12
Labrafil M 1944 CS	22.59 ± 1.66	9

* Hydrophilic-lipophilic balance of the vehicles; values obtained from Gatefosse website [29].

**Table 2:** Design of experiments (DoE) of liquid butenafine self-nanoemulsifying drug delivery systems (B-SNEDDS). Excipient quantities are expressed as a fraction, considering that the sum of all excipients was equal to 1 g. For each combination, the average particle size (*n* = 3) in numbers after dilution in de-ionised water (1:1000 *w*/*w*) was illustrated.

Experiment	Labrasol	Capryol 90	Peceol	Particle Size (nm)
1	0.29	0.50	0.2	116
2	0	0.50	0.49	376
3	0	0.8	0.2	289
4	0.19	0.2	0.60	191
5	0.22	0.41	0.35	156
6	0.29	0.50	0.2	178
7	0.09	0.61	0.29	244
8	0	0.2	0.8	1735
9	0	0.50	0.49	410
10	0.39	0.34	0.26	107
11	0.4	0.2	0.4	101
12	0.12	0.35	0.52	167
13	0	0.2	0.8	1340
14	0	0.34	0.65	1180
15	0	0.8	0.2	352
16	0.19	0.2	0.60	141

**Table 3:** Characterisation of the solid B-SNEDDS. Results are reported as mean ± SD (*n* = 3).

Code	Aerosil Type	Carrier: B-SNEDDS Ratio (*w*/*w*)	Yield (%)	Angle of Repose (°)	Drug Loading (%)	Particle Size (nm)	Butenafine Released at 60 min in SGF (%)
F1	200	1:2	71.1 ± 1.6	19.1 ± 5.4	94.1 ± 0.1	119.8 ± 2.3	45 ± 2.0
F2	200	1:3	66.5 ±10.2	15.9 ± 3.0	96.5 ± 2.4	25.9 ± 1.2	28 ± 5.0
F3	R972	1:2	32.9 ± 1.3	20.6 ± 0.2	85.5 ± 4.6	279 ± 8.7	18 ± 4.0
F4	R972	1:3	47.4 ± 22.6	8.7 ± 1.5	97.0 ± 2.1	1 *	20 ± 0.2

* Particle size measurements of F4 were registered as 1 nm, indicating that particles were not stable upon dilution.

**Table 4:** Hardness of the F1 solid B-SNEDDS formulation. Hardness expressed as minimum and maximum values.

Compaction Pressure (kN)	Hardness (N)	Dimension (mm)
9806.65	15.6–18.4	12.91 × 3.17
19,613.3	7.7–20.1	12.91 × 3.17

**Table 5:** Antileishmanial activity of solid B-SNEDDS formulations and butenafine were assayed against promastigote and amastigote forms of *L. (L.) infantum*. Cytotoxicity was analysed using peritoneal macrophages from BALB/c mice. EC_50_ represents the concentration of the formulation that produced a 50% reduction in parasites, while CC_50_ represents the concentration of the formulation that produced a 50% reduction of cell viability in treated culture cells with respect to untreated ones. SIp: selectivity index towards promastigotes forms; SIa: selectivity index towards amastigotes forms.

Formulation	EC_50_ (µM) Promastigote	CC_50_ (µM) Macrophage	EC_50_ (µM) Amastigote	SIp	SIa
F1	76.5 ± 3.0 *	225.8 ± 3.2	164.4 ± 3.4	2.9	1.3
F2	93.1 ± 3.0	127.2 ± 3.0	702.5 ± 3.2	1.3	0.1
F3	101.7 ± 3.2	≥300.0	86.4 ± 2.9 *	≥3.0	≥3.6
F4	73.9 ± 3.2 *	233.1 ± 3.0	27.0 ± 3.4 *	3.1	8.6
Butenafine	99.8 ± 3.1	109.3 ± 3.5	118.4 ± 3.6	1.0	0.9
Miltefosine	17.9 ± 0.9	126.3 ± 3.5	13.7 ± 0.7	7.0	9.2

* *p* ≤ 0.05 compared to Butenafine.

## References

[1] Araújo-Santos T., Prates D.B., Andrade B.B., Nascimento D.O., Clarêncio J., Entringer P.F., Carneiro A.B., Silva-Neto M.A., Miranda J.C., Brodskyn C.I. (2010). Lutzomyia longipalpis saliva triggers lipid body formation and prostaglandin E₂ production in murine macrophages. PLoS Negl. Trop. Dis..

[2] World Health Organization (WHO) Leishmaniasis Disease and Epidemiology. http://www.who.int/leishmaniasis/epidemic/response_more/en/index.html.

[3] Rittig M.G., Bogdan C. (2000). Leishmania-Host-cell Interaction: Complexities and Alternative Views. Parasitol. Today.

[4] Laurenti M.D., Da Matta V.L.R., Pernichelli T., Secundino N.F.C., Pinto L.C., Corbett C.E.P., Pimenta P.P.F. (2009). Effects of Salivary Gland Homogenate from Wild-Caught and Laboratory-Reared Lutzomyia longipalpison the Evolution and Immunomodulation of Leishmania (Leishmania) amazonensis Infection. Scand. J. Immunol..

[5] Laison R., Shaw J.J. (1988). New world Leishmaniasis—The Neotropical Leishmania species. Topley & Wilson. Microbiology and Microbial Infections.

[6] Silveira F.T., Lainson R., Crescente J.Â., De Souza A.A., Campos M.B., Gomes C.M., Laurenti M.D., Corbett C.E. (2010). A prospective study on the dynamics of the clinical and immunological evolution of human Leishmania (L.) infantum chagasi infection in the Brazilian Amazon region. Trans. R. Soc. Trop. Med. Hyg..

[7] Dantas-Torres F. (2006). Leishmania infantum versus Leishmania chagasi: Do not forget the law of priority. Memórias do Instituto Oswaldo Cruz.

[8] Barak E., Amin-Spector S., Gerliak E., Goyard S., Holland N., Zilberstein D. (2005). Differentiation of Leishmania donovani in host-free system: Analysis of signal perception and response. Mol. Biochem. Parasitol..

[9] Santos D.O., Coutinho C.E.R., Madeira M.F., Bottino C.G., Vieira R.T., Nascimento S.B., Bernardino A.M.R., Bourguignon S.C., Côrte-Real S., Pinho R.T. (2008). Leishmaniasis treatment—A challenge that remains: A review. Parasitol. Res..

[10] Kaur G., Rajput B. (2014). Comparative Analysis of the Omics Technologies Used to Study Antimonial, Amphotericin B, and Pentamidine Resistance in Leishmania. J. Parasitol. Res..

[11] Serrano D.R., Lalatsa A. (2017). Oral amphotericin B: The journey from bench to market. J. Drug Deliv. Sci. Technol..

[12] Smith L., Serrano D.R., Mauger M., Bolás-Fernández F., Dea-Ayuela M.A., Lalatsa A. (2018). Orally Bioavailable and Effective Buparvaquone Lipid-Based Nanomedicines for Visceral Leishmaniasis. Mol. Pharm..

[13] Fernández O.L., Díaz-Toro Y., Ovalle C., Valderrama L., Muvdi S., Rodriguez I., Gomez M.A., Saravia N.G. (2014). Miltefosine and Antimonial Drug Susceptibility of Leishmania Viannia Species and Populations in Regions of High Transmission in Colombia. PLoS Negl. Trop. Dis..

[14] Dorlo T.P.C., Balasegaram M., Beijnen J.H., De Vries P.J. (2012). Miltefosine: A review of its pharmacology and therapeutic efficacy in the treatment of leishmaniasis. J. Antimicrob. Chemother..

[15] Pérez-Victoria F.J., Sánchez-Cañete M.P., Seifert K., Croft S.L., Sundar S., Castanys S., Gamarro F. (2006). Mechanisms of experimental resistance of Leishmania to miltefosine: Implications for clinical use. Drug Resist. Updates.

[16] Drugs for Neglected Diseases initiative: DNDi. https://www.dndi.org/diseases-projects/leishmaniasis/tpp-vl.

[17] Graul A., Sorbera L., Pina P., Tell M., Cruces E., Rosa E., Stringer M., Castaner R., Revel L. (2010). The year’s new drugs & biologics–2009. Drug News Perspect..

[18] Tobinick E.L. (2009). The value of drug repositioning in the current pharmaceutical market. Drug News Perspect..

[19] Voss A., Soto J., Toledo J., Nicholls R.S., Padilla J., Engel J., Fischer C., Gutierrez P., Berman J. (2001). Treatment of American Cutaneous Leishmaniasis with Miltefosine, an Oral Agent. Clin. Infect. Dis..

[20] Bezerra-Souza A., Yamamoto E.S., Laurenti M.D., Ribeiro S.P., Passero L.F.D. (2016). The antifungal compound 25butenafine eliminates promastigote and amastigote forms of Leishmania (Leishmania) amazonensis and Leishmania (Viannia) braziliensis. Parasitol. Int..

[21] Food and Drug Administration (FDA) Review and Evaluation of Pharmacology/Toxicology Data O Fbutenafine, NDA 21-307 (000)/09-29-2000. https://www.accessdata.fda.gov/drugsatfda_docs/nda/2001/21-307_Lotrimin_pharmr.pdf.

[22] Serrano D.R., Lalatsa A., Dea-Ayuela M.A. (2017). Engineering Synergistically Active and Bioavailable Cost-effective Medicines for Neglected Tropical Diseases; The Role of Excipients. Curr. Top. Med. Chem..

[23] Lavoie F., Cartilier L., Thibert R. (2002). New Methods Characterizing Avalanche Behavior to Determine Powder Flow. Pharm. Res..

[24] Betatek Inc. (2012). Instrumentation Superbly Supported: Microtrac Zetatrac Nanotechnology, Particle Size and Charge Measurement Analyzer.

[25] United States Pharmacopeia (USP) (2008). USP 41-NF36.

[26] Drug Bank Database. https://www.drugbank.ca/drugs/DB01091.

[27] European Pharmacopeia. https://www.edqm.eu/en/european-pharmacopoeia-ph-eur-9th-edition..

[28] Passero L.F.D., Sacomori J.V., Tomokane T.Y., Corbett C.E.P., Da Silveira F.T., Laurenti M.D. (2009). Ex vivo and in vivo biological behavior of Leishmania (Viannia) shawi. Parasitol. Res..

[29] Gattefossé: Excipients for Solubility and Bioavailability Enhancement. https://www.gattefosse.com/excipients-for-solubility-and-bioavailability-enhancement.

[30] Maher S., Brayden D.J., Casettari L., Illum L. (2019). Application of Permeation Enhancers in Oral Delivery of Macromolecules: An Update. Pharmaceutics.

[31] United States Pharmacopeia (USP) (2006). USP 41-NF36.

[32] Goh H.P., Heng P.W.S., Liew C.V. (2018). Comparative evaluation of powder flow parameters with reference to particle size and shape. Int. J. Pharm..

[33] Porter C.J.H., Pouton C.W., Cuine J.F., Charman W.N. (2008). Enhancing intestinal drug solubilization using lipid-based delivery systems. Adv. Drug Deliv. Rev..

[34] Humberstone A.J., Charman W.N. (1997). Lipid-based vehicles for the oral delivery of poorly water soluble drugs. Adv. Drug Deliv. Rev..

[35] Drugbank Butenafine. https://www.drugbank.ca/drugs/DB01091.

[36] Seifert K., Escobar P., Croft S.L. (2010). In vitro activity of anti-leishmanial drugs against Leishmania donovani is host cell dependent. J. Antimicrob. Chemother..

[37] Stütz A. (1988). Synthesis and Structure—Activity Correlations within Allylamine Antimycotics. Ann. N. Y. Acad. Sci..

[38] Ryder N.S. (1992). Terbinafine: Mode of action and properties of the squalene epoxidase inhibition. Br. J. Dermatol..

[39] Roberts C.W., McLeod R., Rice D.W., Ginger M., Chance M.L., Goad L.J. (2003). Fatty acid and sterol metabolism: Potential antimicrobial targets in apicomplexan and trypanosomatid parasitic protozoa. Mol. Biochem. Parasitol..

[40] Singal A. (2008). Butenafine and superficial mycoses: Current status. Expert Opin. Drug Metab. Toxicol..

[41] Tatham L.M., Rannard S.P., Owen A. (2015). Nanoformulation strategies for the enhanced oral bioavailability of antiretroviral therapeutics. Ther. Deliv..

[42] Anton N., Vandamme T.F. (2011). Nano-emulsions and Micro-emulsions: Clarifications of the Critical Differences. Pharm. Res..

[43] Leonaviciute G., Adamovic N.T., Lam H.T., Rohrer J., Partenhauser A., Bernkop-Schnürch A. (2017). Self-emulsifying drug delivery systems (SEDDS): Proof-of-concept how to make them mucoadhesive. Eur. J. Pharm. Biopharm..

[44] Imada C., Takahashi T., Kuramoto M., Masuda K., Ogawara K., Sato A., Wataya Y., Kim H.S., Higaki K. (2015). Improvement of Oral Bioavailability of N-251, a Novel Antimalarial Drug, by Increasing Lymphatic Transport with Long-Chain Fatty Acid-Based Self-Nanoemulsifying Drug Delivery System. Pharm. Res..

[45] Khan A.A., Mudassir J., Mohtar N., Darwis Y. (2013). Advanced drug delivery to the lymphatic system: Lipid-based nanoformulations. Int. J. Nanomed..

[46] Gosh S., Roy T. (2014). Nanoparticulate drug-delivery systems: Lymphatic uptake and its gastrointestinal application. J. Appl. Pharm. Sci..

[47] Elnaggar Y.S., El-Massik M.A., Abdallah O.Y. (2009). Self-nanoemulsifying drug delivery systems of tamoxifen citrate: Design and optimization. Int. J. Pharm..

[48] Shen S., Wu Y., Liu Y., Wu D. (2017). High drug-loading nanomedicines: Progress, current status, and prospects. Int. J. Nanomed..

[49] Serrano D.R., Hernández L., Fleire L., González-Alvarez I., Montoya A., Ballesteros M.P., Dea-Ayuela M.A., Miró G., Bolás-Fernández F., Torrado J.J. (2013). Hemolytic and pharmacokinetic studies of liposomal and particulate amphotericin B formulations. Int. J. Pharm..

